# Cigarette Smoke Exposure during Pregnancy Alters Fetomaternal Cell Trafficking Leading to Retention of Microchimeric Cells in the Maternal Lung

**DOI:** 10.1371/journal.pone.0088285

**Published:** 2014-05-15

**Authors:** Anja Vogelgesang, Cristina Scapin, Caroline Barone, Elaine Tam, Anna Blumental Perry, Christiane E. L. Dammann

**Affiliations:** 1 Division of Newborn Medicine, Department of Pediatrics, Floating Hospital for Children at Tufts Medical Center, Boston, Massachusetts, United States of America; 2 Hanover Medical School, Hanover, Germany; 3 Department of Surgery, Tufts Medical Center, Boston, Massachusetts, United States of America; 4 Sackler School of Graduate Biomedical Sciences, Tufts University School of Medicine, Boston, Massachusetts, United States of America; 5 Genetic and Cellular Biology Division, Dibit. San Raffaele Scientific Institute, Milan, Italy; 6 Department of Biomedical Sciences, Mercer School of Medicine and Department of Laboratory Oncology Research, Anderson Cancer Institute, Memorial University Medical Center, Savannah, Georgia, United States of America; Xavier Bichat Medical School, INSERM-CNRS - Université Paris Diderot, France

## Abstract

Cigarette smoke exposure causes chronic oxidative lung damage. During pregnancy, fetal microchimeric cells traffic to the mother. Their numbers are increased at the site of acute injury. We hypothesized that milder chronic diffuse smoke injury would attract fetal cells to maternal lungs. We used a green-fluorescent-protein (GFP) mouse model to study the effects of cigarette smoke exposure on fetomaternal cell trafficking. Wild-type female mice were exposed to cigarette smoke for about 4 weeks and bred with homozygote GFP males. Cigarette smoke exposure continued until lungs were harvested and analyzed. Exposure to cigarette smoke led to macrophage accumulation in the maternal lung and significantly lower fetal weights. Cigarette smoke exposure influenced fetomaternal cell trafficking. It was associated with retention of GFP-positive fetal cells in the maternal lung and a significant reduction of fetal cells in maternal livers at gestational day 18, when fetomaternal cell trafficking peaks in the mouse model. Cells quickly clear postpartum, leaving only a few, difficult to detect, persisting microchimeric cells behind. In our study, we confirmed the postpartum clearance of cells in the maternal lungs, with no significant difference in both groups. We conclude that in the mouse model, cigarette smoke exposure during pregnancy leads to a retention of fetal microchimeric cells in the maternal lung, the site of injury. Further studies will be needed to elucidate the effect of cigarette smoke exposure on the phenotypic characteristics and function of these fetal microchimeric cells, and confirm its course in cigarette smoke exposure in humans.

## Introduction

Each year, more than 443,000 Americans die of tobacco-related illnesses such as chronic obstructive pulmonary disease (COPD) and lung cancer [1]. These illnesses cause more deaths in women than breast cancer does. Smoking during pregnancy is still a widespread problem. About 14% of women in the United States state that they are smoking during pregnancy [2]. Cigarette smoke (CS) exposure causes oxidative damage to lung tissue and an impairment of the capacity of alveolar macrophages to clear bacteria and apoptotic cells [3], contributing to chronic lung injury in smoking mothers. CS exposure during pregnancy is associated with preterm delivery, low birth weight, and an increased morbidity in the newborn [4]. Animal studies have shown that fetal nicotine exposure may affect the development of the lungs and multiple other organs, and cause long-lasting effects on body adiposity and endocrine function [5].

During pregnancy, fetal cells enter the maternal circulation and are detected as microchimeric cells [6]. Some of these cells persist through one's life [7], but their distribution, impact on maternal health, and preferred organ of residence is not clearly understood. Their presence might be acutely beneficial for the repair of damaged tissue due to their potential stem cell-like properties, but they might also contribute to the development of autoimmune diseases and cancer in women after a certain number of years [8]. In the mouse models, it has been shown that fetal chimeric cells are found in the maternal circulation peaking towards the end of pregnancy. Most of these microchimeric cells are cleared around postpartum day (PD)5–6. The highest number of these cells has been found in the maternal lungs, their likely port of entry [9]. The chimeric cells represent a mixed population of cells, some with stem cell-like properties, and some with more differentiated characteristics [10]. It has been shown that fetal chimeric cells are attracted to the site of severe acute injury [11] and that overall cell trafficking is promoted during loss of pregnancy [12]. The intensity of microchimeric cell trafficking is influenced by the genetic background [13], but both the intensity and the distribution of microchimeric cells in milder chronic, more diffuse injury settings is unclear.

We hypothesized that chronic CS exposure during pregnancy affects trafficking, attraction, and distribution of fetal chimeric cells. Here we present the effects of a mild chronic diffuse lung injury on fetomaternal cell trafficking using a green-fluorescent-protein (GFP) mouse model.

## Materials and Methods

### Mice and cigarette smoke experiments

**Figure 1:**
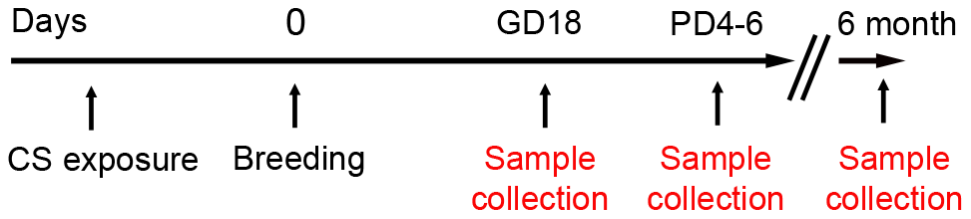
Cigarette smoke exposure model. Cigarette smoke exposure model: After approximately 4 weeks of CS exposure, wild-type female mice were bred with GFP-homozygous males to generate GFP heterozygous fetuses. Pregnant and non-pregnant control females continued to smoke and were sacrificed at gestational day (GD)18, at postpartum day (PD)4–6 and after 6 month. Maternal lungs and livers (GD 18 only) were analyzed for the presence of inflammation and GFP-positive cells.

All animal use was approved by the Institutional Animal Care and Use Committee of Tufts University and Tufts Medical Center (Assurance number: A3775-01). Female wild-type (wt) C57BL/6 mice were obtained from Jackson Laboratories (Bar Harbor, ME, USA); male homozygous C57BL/6-Tg (CAG-EGFP) C14-Y01-FM131Osb Mice were from Riken BioResource Center (Ibaraki, Japan). Eight-week-old female mice were exposed to CS (1R5F research cigarettes, Tobacco and Health Research Institute, University of Kentucky, Lexington, KY, USA) using a standard smoking machine, as previously published [14]. Intake was slowly increased to an exposure of four cigarettes per day for five days per week for approximately 15–46 days (33 days in average) prior to successful mating. CS-exposed wt mice were mated with homozygous GFP-positive C57BL/6 males to generate GFP-heterozygous fetuses. CS exposure was continued until mice were sacrificed (Figure 1). Non smoke-exposed control animals were mated similarly and used as controls. Pregnant mice as well as non-pregnant control mice (smokers and non-smokers) were anesthetized with ketamine (10 mg/ml) on gestational day (GD)18 of pregnancy or postpartum (postnatal day (PD)4–6, or 6 month after pregnancy). Lungs were perfused free of blood from the right ventricle with 10 mL ice-cold phosphate buffered saline. The trachea was exposed and the left lung was ligated, cut off, frozen on dry ice, and stored at −80°C for DNA isolation. The remaining right lung was either injected intra-tracheally with 2 ml of dispase (undiluted liquid; BD, Bedford, MA, USA, cat no: 354235) and 1% low-melt agarose (Bio-Rad, Hercules, CA, USA, cat no: 161–3111) for fluorescent activated cell sorting (FACS) analysis or inflated with 10% neutral buffered formalin at a constant pressure of 25 cm H_2_O for 10 minutes. After inflation, the trachea was ligated and the inflated lungs were removed, fixed in formalin for 24 hours, parted in lobes, and transferred into phosphate buffered saline for later paraffin embedding and cutting. Livers were frozen at -80°C for DNA analysis.

### Mac3 immunohistochemistry staining

To identify alveolar macrophages, lung sections were stained with macrophage specific Mac3. Sections were melted and washed with xylene and ethanol. Antigen retrieval was performed in a steamer with Target Retrieval Solution (Dako, Carpinteria, CA, USA, cat no: S1699) for 30 minutes. Slides were blocked with Peroxidase Blocking Reagent (Dako, cat no: S2001) and 5% goat serum, followed by incubation in Avidin/Biotin Block (Vector, Burlingame, CA, USA, cat no: SP-2001) for 15 minutes each. Staining with Mac3 antibody (rat anti-mouse Mac3 antibody; BD Pharmingen, San Diego, CA, USA, cat no: 550292) was performed overnight at 4°C. After staining with the biotin-conjugated goat anti-rat IgG (Vector, cat-no: BA-4000) for 2 hours at 4°C, an additional peroxidase blocking was performed with 3% H_2_0_2_ in MeOH for 10 minutes, which was followed by incubation with ABC reagent (Vector, cat no: PK-6100) for 30 minutes. Sections were incubated in diaminobenzidine solution (DAB Peroxidase Substrate Kit, 3,3′- diaminobenzidine; Vector, cat no: SK-4100) until desired stain intensity developed. They were then rinsed in tap water, counterstained with hematoxylin (Mayer's Hematoxylin; Rowley Biochemical, Danvers, MA, USA, cat no: L-756-1A), rinsed, dehydrated in ascending series of ethanol, and finally mounted in Cytoseal (Cytoseal-60; Fisher Scientific, Ottawa, ON, USA, cat no: 23-244-257). Macrophages were counted and numbers normalized per mm lung tissue using lung morphometry as described in Shapiro et al [15]. Airspace was quantified by average Chord Length measurements obtained using a previously published, automated image processing algorithm, and Scion image processing software (National Institutes of Health). The software program automatically created a binary black and white image by separating original images into tissue and airspace after large airways, blood vessels, and other nonalveolar structures were manually removed from the images. Pre-drawn horizontal and vertical grids of one pixel width were laid over the individual image. Lines ending on or intercepting with alveolar tissue were counted by analyzing the number of pixels in every object. The image only included lines of one pixel width so that this measurement served as a length. According to methods adapted from Dunhill, this number was used to calculate the average Chord Length [16]. The amount of macrophages was normalized to millimeter of alveolar wall using the following formula: 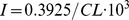



CL: Chord Length (mean value given by Excel during morphometry)

I: Amount of macrophages per mm lung tissue

(10^3^ because CL is in µm and I is in mm).

### GFP immunohistochemistry staining

Immunostaining with GFP antibodies was performed in frozen lung sections. The primary antibody has successfully been used for GFP detection before [17]. The procedure and reagents were similar to the immunostaining of macrophages with the following exceptions: After fixation of frozen sections in pre-cooled acetone, antigen retrieval was performed for 10 minutes. The primary antibody (rabbit anti-GFP IgG; Invitrogen, Grand Island, NY, USA, cat no: A11122) was incubated for 1 hour at 37°C, followed by staining with the biotinylated goat anti-rabbit antibody for 30 minutes at 37°C. During incubation with diaminobenzidine, the desired diaminobenzidine intensity was checked under the microscope using the lung section of a GFP-positive animal.

### GFP immunofluorescence

After fixation in pre-cooled acetone, nuclear staining was performed with Hoechst (Bisbenzamide; Sigma-Aldrich, St. Louis, MO, USA, cat no: B2883) for 30 seconds and slides were mounted. Cryostat sections of mice at GD18 and non-pregnant controls were evaluated for GFP-positive cells with a confocal laser scanning microscope (TCS SP2, Leica).

### Flow cytometry of GFP-labeled cells

To evaluate the number of fetal cells entering the maternal lungs during pregnancy, the number of GFP-positive cells was analyzed in maternal lung tissue. Right lungs were removed, minced, and incubated in dispase/collagenase (final concentration: 2 mg/ml; Roche Applied Sciences, Indianapolis, IN, USA, cat no: 10269638001) in PBS for 45 minutes at 37°C. 0.025 mg/ml DNase (Sigma-Aldrich, St. Louis, MO, USA, cat no: D4527) was added for 4 minutes on ice and digested tissue was filtered through 100 µm and 40 µm filters. Cells were resuspended in 3% paraformaldehyde, fixed for 20 minutes at 4°C, and analyzed in 10% fetal bovine serum in phosphate buffered saline by FACS using a Cytomation MoFlo (Beckman Coulter, Indianapolis, IN, USA). Data of the whole cell suspension were collected and analyzed for GFP-fluorescence based on FL1 (Argon laser for excitation at 488 nm). Data were analyzed with Cytomation Summit software (Cytomation, Fort Collins, CO, USA). Gating was performed according to the controls. Lungs of virgin wild-type female C57BL/6 mice were used as negative controls and lungs of non-exposed GFP-positive pups (GD18) were used as GFP-positive controls. Data were presented as total number of GFP-positive cells per right lung.

### Quantitative real-time PCR (qPCR)

Genomic DNA was extracted using the Qiagen DNeasy Blood and Tissue kit (Qiagen, Valencia, CA, USA, cat-no: 69504) following the manufacturer's instructions. DNA elution was performed twice using 100 µl H20 followed by 100 µl AE buffer. The DNA yield was quantified with a spectrometer and DNA stored at 4°C until further analysis. 600 ng DNA was loaded per well for GFP and 100 ng for Apolipoprotein B (ApoB) analysis in a total volume of 50 µl, as previously published [18]. Pan et al have shown that in a titration curve using GFP-concentrations of 100% to 0.001% in a total of 1000 ng DNA the Threshold Cycle Number (Ct) for ApoB remained constant, while the Ct for GFP showed an appropriate decrease in the concentration of GFP. When loading serial dilutions (from 1∶1 to 1∶100,000) of GFP-positive DNA with one GFP copy per cell, the Delta of the Threshold Cycle Number (DCt) remained constant, which indicates that the PCR efficiency for GFP and ApoB is similar. These authors finally used 1000 ng of DNA for all further experiments compared, while we have, similar to Fujiki *et al*, used 600 ng [9]. Each sample was loaded in triplicates. PCR-Primers and FAM labeled probes were designed and synthesized by Applied Biosystems (Carlsbad, CA, USA). The following primers and probe sequences were used for GFP: Forward primer: 5′ -TGCTGCTGCCCGACAA-3′, reverse primer: 5′ -TGTGATCGCGCTTCTCGTT-3′, Taqman probe: 5′ - FAM-CCACTACCTGAGCACCC-3′ [11]. Apolipoprotein B (ApoB) was used as an internal control to measure the total content of DNA. Forward primer: 5′-CGTGGGCTCCAGCATTCTA-3′, reverse primer: 5′-TCACCAGTCATTTCTGCCTTTG-3′, Taqman probe: 5′-FAM-CCTTGAGCAGTGCCCGACCATTC-TAMRA-3′ [19]. Real-Time qPCR analysis was performed using the Applied Biosystems, 7900 HT FAST Real-Time PCR system and SDS2.2.2 software for analysis. Thermal profile: First cycle: 2 minutes at 50°C, 10 minutes at 95°C, 40 cycles: 15 seconds at 95°C, and 1 minute at 60°C. Each threshold cycle number was determined as the first cycle at which a threshold value in fluorescence signal was reached. Single difference in threshold cycle (DCt) values were calculated for GFP by subtracting the Ct of ApoB from the cycle of GFP. Each sample was loaded as triplicates so that averages of Ct values were used. DDCt values of CS-exposed samples were determined by subtracting the mean of the DCt of the controls of that specific PCR run from the mean of the CS-exposed sample's DCt. The DDCt value expresses the difference of the content of GFP in CS-exposed samples compared to controls. A negative value indicates a smaller DCt, which means that the threshold of the GFP fluorescent signal is reached in an earlier cycle assuming a constant ApoB signal. To better understand if the increased number of GFP positive cells found in maternal lungs were a result of an overall increase in trafficking of microchimeric cells or a redistribution of trafficking fetal cells towards the injured maternal lung, we also performed additional qPCR of maternal livers at GD18. DNA of liver tissue was isolated and processed similarly to lung samples.

### Statistics

For the statistical analysis, unpaired two-tailed or one-tailed t-tests were used where appropriate. All data were presented as mean ± standard error of the mean (SEM) unless otherwise stated.

## Results

### Validation of CS exposure model in pregnant mice

#### A) CS exposure results in inflammation of and an increased number of macrophages in maternal lungs

**Figure 2:**
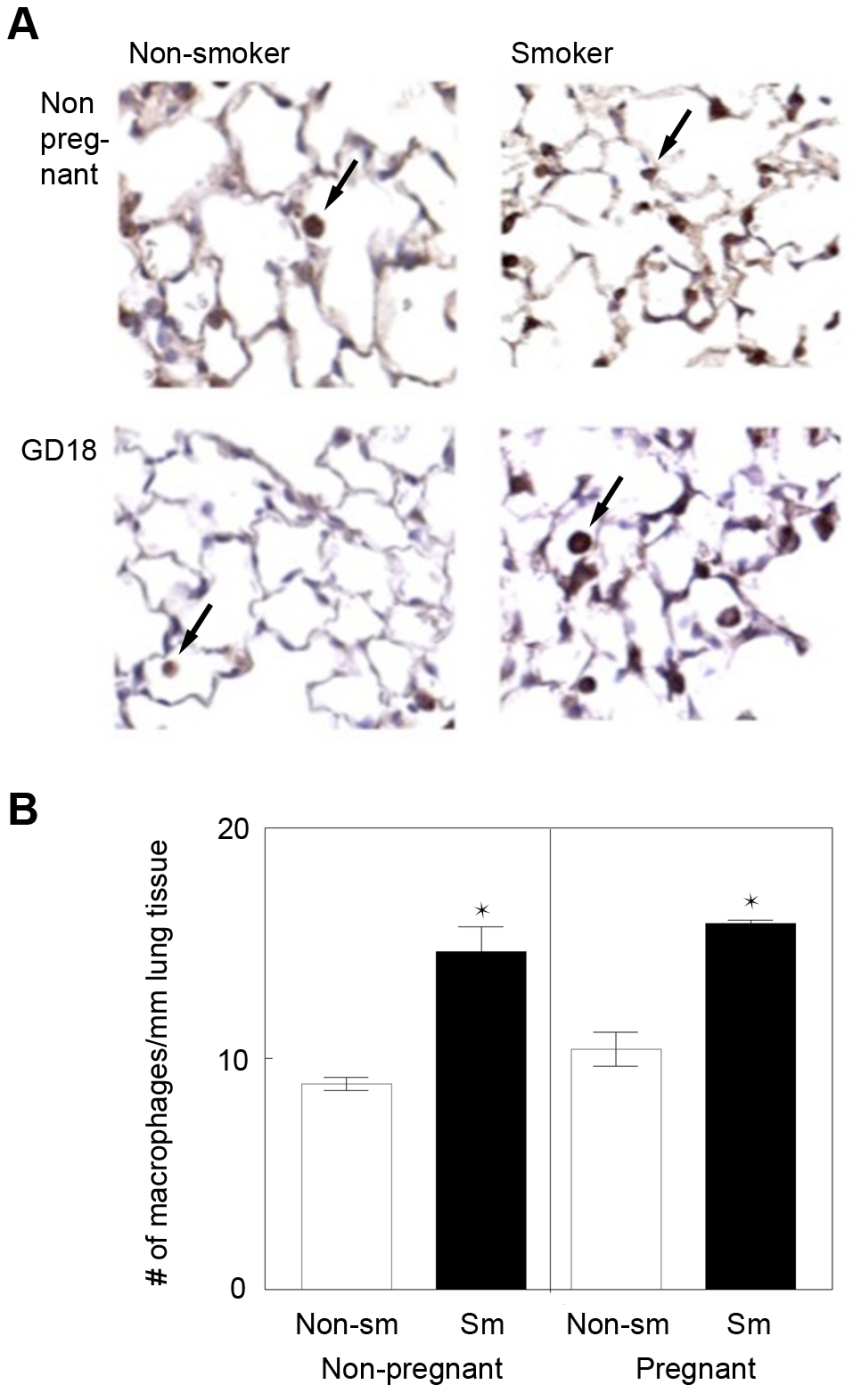
CS exposure increases macrophages in maternal lungs. (A): Immunohistochemical staining in non-pregnant (upper panel) non-smoking (left) and smoking females (right) and in gestational day (GD) 18 pregnant (lower panel) non-smoking (left) and smoking (right) females. Original magnification is 200×. Arrows point to alveolar macrophages. (B): For quantification, macrophages were counted and normalized per mm lung tissue. CS-exposed samples were compared to non-exposed controls (*p<0.05, mean ± SEM, n = 2–4).

It has been shown that inhalation of CS leads to cell injury, cell death, and inflammation with recruitment of neutrophils, macrophages, and T cells. Inhalation of CS also results in impaired activity of inflammatory cells, leading to slow, progressive lung destruction [14]. In mouse models of CS exposure, macrophage accumulation can be detected as early as 4 weeks. We evaluated macrophage recruitment in parasagittal lung sections obtained from smoker and control animals about 6 weeks after starting the CS exposure, using immunohistochemistry staining for Mac3, an antigen expressed in macrophages (Figure 2A). The number of Mac3-positive cells was calculated per mm of the alveolar wall using a published morphometric analysis[14]. CS exposure resulted in a significant increase in the number of macrophages in the lungs independent of pregnancy (Figure 2B). Mean number of macrophages per mm of alveolar wall was 15±1.09 in non-pregnant smokers (n = 3) and 16±0.16 in pregnant smokers (n = 2). In lungs of non CS-exposed mice, the mean number of macrophages was 9±0.29 in non-pregnant (n = 4) and 10±0.74 in pregnant (n = 3) mice. The increase of macrophages in smokers compared to non-smokers was statistically significant for both groups non-pregnant and pregnant (non-pregnant smokers versus non-pregnant non-smokers: p = 0.0284, pregnant smokers versus pregnant non-smokers: p = 0.0187). Again, there were no further differences in the macrophage numbers between pregnant and non-pregnant smokers (non-pregnant versus pregnant non-smokers: p = 0.1994, non-pregnant versus pregnant smokers: p = 0.3866) indicating that the duration of CS exposure or of the presence of fetal microchimeric cells was too short to see a significant impact on macrophage numbers. In summary, our results confirm that our CS exposure injury model produced an inflammation in lungs of smokers.

#### B) CS exposure leads to lower weight in newborn mice

**Figure 3:**
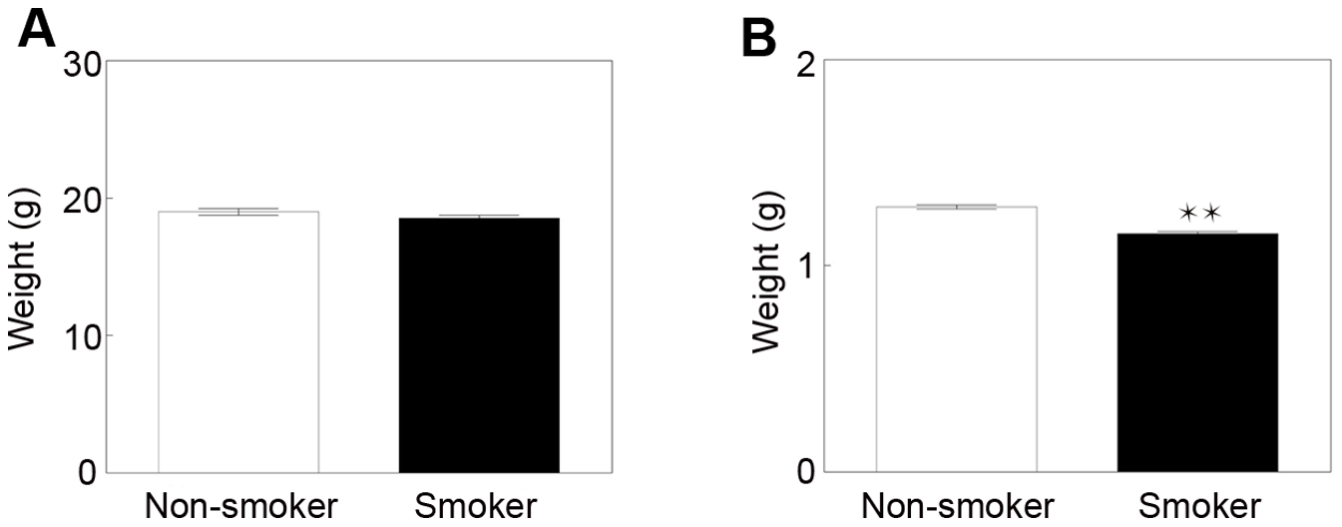
CS exposure decreases weights in the offspring. (A): CS exposure did not reduce weight of adult virgin mice (mean ± SEM, n = 19–31). (B): CS exposure significantly reduced weights of offspring at birth (**p<0.0001, mean ± SEM, n = 49–81).

CS exposure is known to affect weight of humans and mice [20], [21], [22]. In our model, a short 3-week CS exposure prior to breeding resulted in a modest 2.6% decrease in weight of smoking mice (18.53±0.22 g, n = 31) compared to non-smoking mice (19.01±0.25 g, n = 19, p = 0.1497). The absence of a statistical significance might be explained by the short duration of CS exposure, leading only to a trend in weight decrease (Figure 3A). Importantly, intrauterine CS exposure led to a significantly lower weight in newborns (1.16 g±0.01 g, n = 49) when compared to the non-exposed littermates (1.29 g±0.01 g, n = 81, p<0.0001) (Figure 3B). Furthermore, we observed that the rate of pregnancies after overnight mating was decreased in CS-exposed mice (17.39%, n = 46) compared to non-exposed controls (23.81%, n = 42). A high number of CS-exposed offspring died (17 out of 63), while none of the non CS-exposed offspring died (total of 106). There was no difference in the number of pups per litter between the CS-exposed litter (mean 7.87, n = 15) and control litter (mean 7.88, n = 24).

### CS exposure during pregnancy leads to a retention of trafficking fetal cells in the maternal lungs at GD18

**Figure 4:**
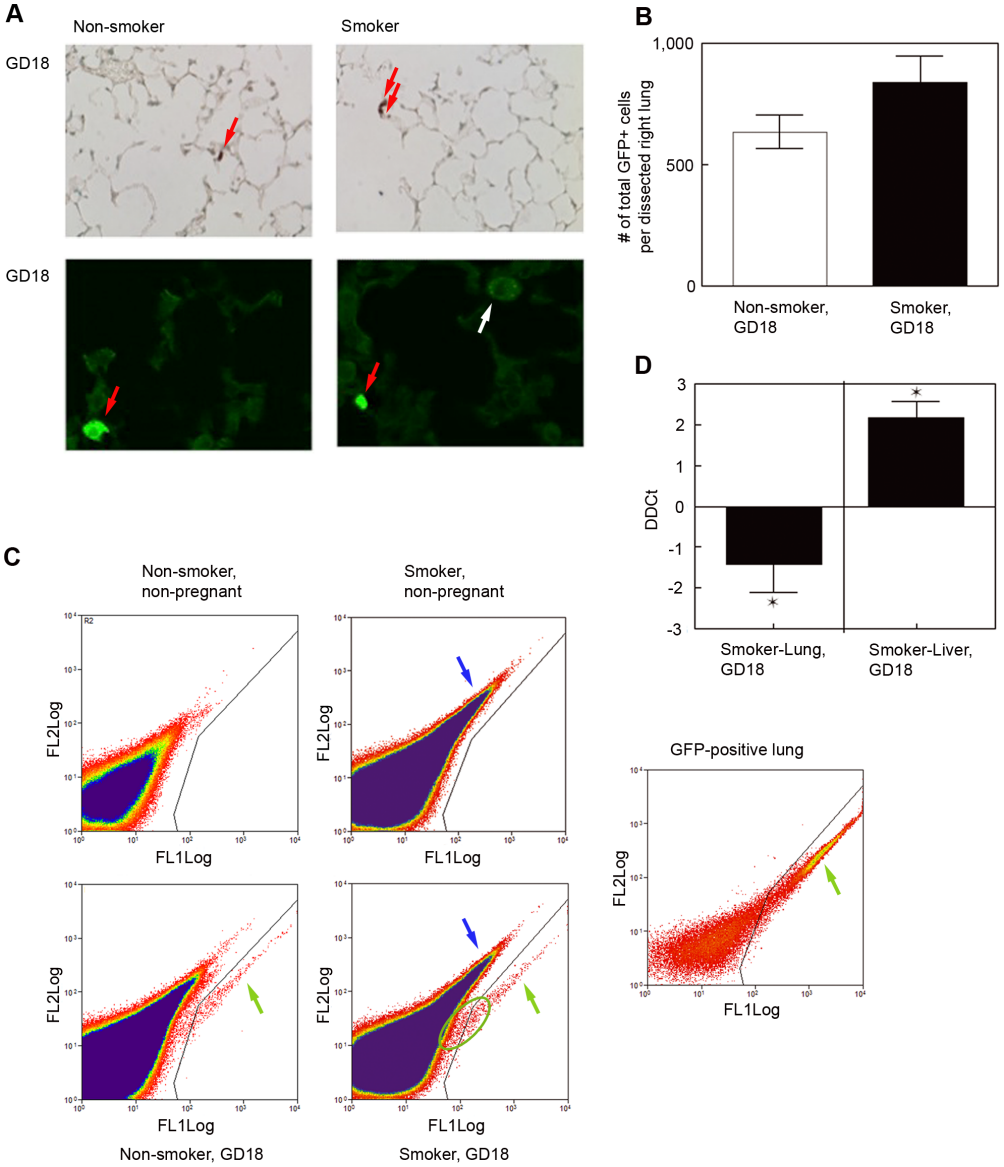
Analysis of fetomaternal trafficking on GD18. (A): Fetal GFP-positive cells were detectable by immunohistochemistry (IHC) (upper panel) and immunofluorescence (IF) (lower panel) at GD18 of pregnancy. For IHC sections primary rabbit anti-GFP IgG was used (magnification is 200×). IF pictures were taken with a confocal microscope. CS-exposed macrophages are known to acquire autofluorescence (white arrow), but they were clearly distinguishable from GFP-positive cells (red arrows). (B–D): GFP-positive cells were quantified by FACS analysis (B, C) and by qPCR analysis (D). (B): Quantification of GFP-positive cells in lungs of pregnant non-smokers and smokers by FACS. Bars represent mean ± SEM of total number of GFP-positive cells included in the gate per dissected right lung (mean ± SEM, n = 4–5). (C): Samples from non-pregnant mice were used as negative (upper graphs), and lungs of GFP-positive pups as positive controls for gating (graph in third column, green arrow points to GFP-positive cells). Samples of CS-exposed mice showed autofluorescent cells (right upper and lower graph, blue arrow), which were easily distinguishable from GFP-positive cells (green arrow). The area included in the ellipse contains potential GFP-positive cells outside of the GFP-positive gate. Those cells were not included in the calculation of the number of GFP-positive cells by FACS shown in B. (D): Quantification of GFP content in maternal lung (left) and liver (right) on GD18 of pregnancy by qPCR. Means of DCt values of non-exposed controls were compared to CS-exposed samples. Results are shown as DDCt values. (lung: *p<0.05 one-sided, mean ± SEM, n = 7) (liver: *p<0.05, mean ± SEM, n = 3–6).

In order to compare the pulmonary retention of fetal cells in non-smoking mothers to smoking mothers, we analyzed the maternal lungs at gestational day (GD)18, when the number of fetal cells in maternal organs is known to be highest [9]. At GD18, fetal chimeric cells were detected in maternal lungs of smokers and non-smokers by immunohistochemical staining, as well as by fluorescence analyses using a confocal microscope (Figure 4A). The fetal GFP-positive cells were easily distinguishable from autofluorescent macrophages in slides of CS-exposed lungs, due to their uninterrupted bright fluorescence in contrast to the irregular fluorescence in the granules of macrophages. The very low number of fetal cells per test field made quantification via this method impossible. We therefore performed FACS analysis in the right side of the maternal lung. A total of 8 to 18 million cells (mean number 12 million cells) were obtained by digesting the right lung into a single cell suspension, which was used for analysis. Quantitative analysis by FACS showed that the number of fetal cells at GD18 was increased in CS-exposed lungs (834±112, n = 5) compared to non-exposed control lungs (633±68, n = 4, p = 0.1756) (Figure 4B). This difference did not reach statistical significance. The actual number of GFP positive cells, identified by FACS, might be slightly underestimated due to their preferred presence on the outskirts of the gate for GFP positivity. Populations of GFP positive cells were seen at the outskirt of the gate, making it difficult to define a clear cut-off between positive and negative cells (Figure 4C). This is a known phenomenon previously reported by others [9]. Since FACS and qPCR analysis are the most common sensitive methods to detect microchimeric cells [23], [24], we confirmed our FACS results with qPCR analyses. qPCR revealed a statistically significant increase in GFP-positive DNA and therefore a negative DDCt value of −1.41±0.71 (n = 7) in CS-exposed maternal lungs when compared to non-exposed lungs (n = 7, p = 0.0469) (Figure 4D). We therefore concluded that CS exposure results in an increased retention of fetal cells in the maternal lungs.

### CS exposure leads to lower numbers of fetal cells in maternal livers at GD18

Intrauterine blood flow directs fetal cells to enter the maternal circulation through the maternal pulmonary arteries, leading to a presumed higher frequency of fetal cells in the maternal lung [9]. Fetal cells travelling to the mother pass through the lungs before reaching other organs [9], [25]. CS injures the lung and increases the retention of microchimeric cells. In order to verify if this phenomenon is due to an increased trafficking or redistribution of microchimeric cells, we analyzed the maternal liver for the number of chimeric cells. We used the more quantitative approach with qPCR analyses. CS exposure led to a significant decrease of GFP-positive DNA (DDCt = 2.17±0.40, n = 6) in the maternal liver at GD18 compared to livers from non CS-exposed control mice (n = 3, p = 0.0028) (Figure 4D). Therefore, we concluded that CS exposure alters the distribution of fetal cells, with a decrease of fetal cells in the maternal liver and their retention in the maternal lungs, which is the site of injury and inflammation.

### CS exposure does not change the postpartum clearance of fetal cells from maternal lungs

**Figure 5:**
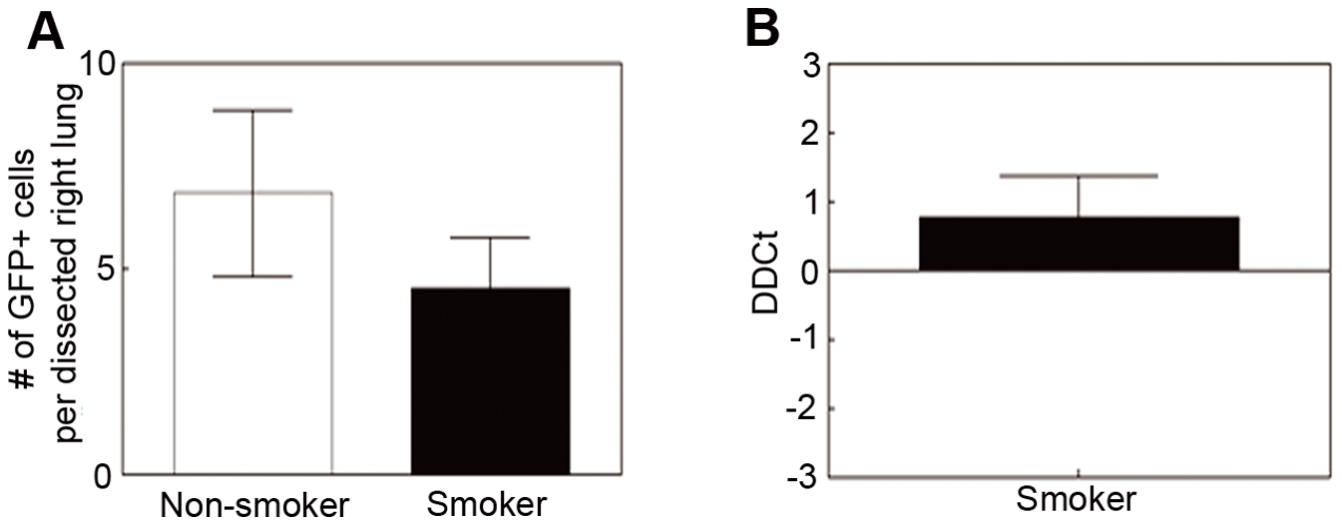
Analysis of postpartum clearance of fetal cells from the maternal lungs. Postpartum clearance of fetal cells is independent of CS measured at PD4–6 by FACS (A) and qPCR analyses (B). Samples were processed and analyzed similarly to GD18 (mean ± SEM, n = 6 (A), n = 5–10 (B)).

To analyze the effect of CS exposure on the clearance rate of fetal cells from the maternal lungs, we studied the presence of fetal cells at postpartum days (PD) 4–6. It is known that most microchimeric cells are cleared at this time point making them almost non-detectable [9]. FACS analysis was performed on 5 million to 26 million cells (mean number 12 million cells). CS did not significantly change the clearance of fetal cells, although there may be a trend towards a faster clearance in lungs of smokers (4.5±1.23, n = 6) compared to non-smokers (6.83±2.02, n = 6, p = 0.3535) (Figure 5A). These FACS results were again confirmed by qPCR analyses. The trend towards faster clearance in CS-exposed lungs (DDCt = 0.79±0.59, n = 5, p = 0.2548), when compared to non CS-exposed control lungs (n = 10) was confirmed by qPCR (Figure 5B); however, neither result reached statistical significance. This might be due to the overall low number of fetal cells at PD4-6 in syngeneic strains (1–10 cells in smokers and 4–15 cells for non-smokers). Indeed, it has been shown that fetal cell trafficking is higher and fetal cells persist longer in mice of allogeneic crossing [26]. In allogeneic crossings of C57BL/6 with transgenic mixed genetic background mice, we found about 90,000 cells on PD6 in the maternal lungs of non-smokers, while less than half that number existed in the CS-exposed lungs (own unpublished data).

In humans, fetal cells have been found in the lungs even 27 years after pregnancy [7]. Therefore, we tested whether GFP-positive cells can be detected long after pregnancy in lungs of mice exposed to CS. Lungs of mice exposed to CS starting one month before pregnancy and ending 6 months after were harvested and analyzed for the presence of GFP-positive cells by FACS analysis, compared to their non-smoking pregnant controls. 8 to 11 million cells (mean number 10 million cells) were counted by MoFlo software as described in Material and Methods. We were unable to detect any GFP-positive cells (n = 4) independent of exposure to CS. We concluded that there is no preferential significant long-term retention of fetal cells in the lungs of syngeneic crossbred mice.

## Discussion

Fetomaternal cell trafficking has been demonstrated in healthy mice [9] and human pregnancies [7]. In mice, it has been demonstrated that trafficking and distribution of fetal cells can be influenced by acute localized injury, attracting fetal cells to the site of injury [11], [27]. It is not known if injury leads to an overall increase in cell trafficking or altered retention of chimeric fetal cells in the injured maternal organs. Very limited information is available on the effects of chronic, relatively mild diffuse injury on fetomaternal cell trafficking. In this study, we used maternal CS exposure, a mild, slowly-progressing diffuse inflammatory injury to the lung, to study its effect on fetomaternal trafficking. First, we validated our CS exposure animal model and confirmed that CS exposure induced lung inflammation as previously seen [3]. The presence of almost double the amount of macrophages in the lungs of smokers compared to non-smokers is indicative of both inflammatory and possible reparative responses triggered by CS exposure. There was no difference in cell number between non-pregnant and pregnant smokers, indicating that pregnancy-induced changes including the presence of microcimeric cells do not affect this inflammatory response after short-term CS exposure. The role of macrophages in CS-induced damage is a focus of vigorous investigation, as these cells secrete numerous proteases that eventually add to CS-induced tissue destruction later in the course of injury. It is assumed that macrophages, which are recruited as M1-phenotypes pro-inflammatory cells in CS, are causing reprogramming of alveolar macrophages into partially M2-polarized macrophages with tissue remodeling properties [28]. But this develops much later in the course of exposure and we here focused our analysis on the days of the known peaks of intrauterine cell trafficking at GD18 and its quick postpartum clearance [9].

Secondly, we confirmed effects of CS exposure on the intrauterine environment, finding lower weights in the offspring of smoking mothers, also reported by others [29]. In addition to the lower weights, we observed a higher incidence of stillbirth in CS-exposed pregnancies. An association between maternal smoking during pregnancy and stillbirth has been shown before in human pregnancies [4].

CS exposure was associated with an increase in the number of fetal cells in the maternal lungs and a significant decrease of cells in the maternal livers at the known peak of naturally occurring microchimerism at GD18. It has been suggested that in mouse models of acute injuries, injury most likely attracts microchimeric cells by altered tissue attraction of exciting cells. Acute cardiac injury leads to selective retention of fetal cells to the maternal heart [30]. This was also shown in a brain injury model, where the overall number of fetal cells was not significantly increased, but cells were attracted to the site of injury [11]. The injury caused by CS develops slowly, is diffuse, and will lead to anatomic lung injury after years of exposure. We here present new insights on this mild chronic diffuse injury model being able to result in retention of microchimeric fetal cells at the site of injury. This is an important discovery, because mild chronic injuries are more common in life than focal acute injuries. The mild chronic injury we induced led only to a small but significant increase in the number of fetal cells in the maternal lung. This is in alignment with studies in humans [31] and mice [32], [33] indicating that the presence of chimerism relates to the extent and degree of injury. Additionally, CS-exposed fetuses are significantly smaller than non-exposed controls, and smaller litter weight is known to result in a smaller number of trafficking fetal cells [9], [10]. Another reason for our small increase in the number of microchimeric cells detected in the maternal lung after CS exposure is the fact that some of these fetal cells might be damaged by CS exposure and might undergo apoptosis following removal by macrophages.

The clinical impact of retention of these fetal cells on an injured organ is not clear. Knowing that fetal microcimeric cells have potential stem cell like properties [34], one could assume that fetal cells can contribute to the repair processes. Indeed, in some cases the fetal progenitors cells have acquired tissue-specific characteristics after entering the maternal organism during pregnancy [34], emphasizing this assumption of their contribution in repair processes. At present, it is not clear if these cells are able to proliferate [35], differentiate [35], [36], [37], or fuse with organ-specific cells [37], [38]. Recent publications have reported that fetal cells seem to differentiate into various cell types [34], [39]. In human breast cancer, fetal cells were found in 9 out of 10 samples, whereas no cells where found in non-diseased control samples [40]. Similarly in human papillary thyroid cancer, 11 fetal cells per 1 million maternal cells were found in the diseased tissue, while only 2 cells per million were detected in non-affected tissue [41]. In autoimmune thyroiditis, human fetal cells were not only found in the diseased tissue [42], but also increased in the peripheral blood [43]. Despite the clear evidence of an increase of fetal microchimerism in affected tissue, the question of its function in the disease processes and its influence on maternal health is as yet unresolved.

Our finding of the short duration in the maternal lung after pregnancy, suggests that CS exposure might have negative adverse effects on survival, regeneration capacity, migratory ability, and the cytokines profiles of these chimeric cells, which has been reported before [3], [44], [45]. The trend towards a faster postpartum clearance of fetal cells from the smoker lungs supports this idea and may reflect a more rapid senescence of these cells in the setting of continuous CS exposure, as has been described for other stem cells in oxidative stress injury [46]. We did not detect fetal cells in the lungs of smokers 6 month after pregnancy. It may be that only a few fetal cells are retained in the lungs of smoking mothers and they are not detectable using our methods. We did not examine possible anti-GFP alloresponse that might be triggered by the mobilization of macrophages and the known T cell immunogenicity of GFP, resulting in a clearance of these GFP cells.

The impact of CS on health is well known. The impact of the retention of fetal cells in the maternal lung and the observation of fewer cells in the maternal liver are very intriguing findings, but its impact is not clear. It can be explained by a few non-exclusive possibilities. First, CS exposure could cause retention of fetal cells at the site of injury as an inconsequential response to tissue damage, decreasing the pool of trafficking cells to other organs. Second, CS exposure may result in altered capacity for movement and retention of damaged fetal cells, keeping them in the lungs, the port of entry. A third possibility is that CS-injured alveolar macrophages cannot remove them. Fourth, CS exposure may affect the ability of fetal cells to migrate efficiently to other organs. Indeed, it has been demonstrated that CS exposure affects the Rho and Rac GTPase balance, which is crucial for efficient cell movements [3], [47]. And fifth, fetal chimeric cells might have originated from the placenta [30], [48]. Considering the effects of maternal CS exposure on placental blood flow and the trophoblasts itself in humans [4], it could be most likely that these fetal cells entering the maternal organism are already affected in their biology, specifically their potential for movement.

A weakness of our study is that we have not been able to study the biology and function of these microchimeric cells or the effects of a longer duration of CS exposure on trafficking. However, to our knowledge, this study is the first to analyze CS exposure in combination with analysis of fetomaternal trafficking. Our research focused on the maternal lung tissue; CS might influence trafficking to other organs. Since the highest median number of chimeric cells have been found in the lungs, liver, and spleen [9], and these numbers are already at the border of detection by FACS and qPCR analyses, analysis of other organs seems almost impossible and even more challenging. In summary we here demonstrate that CS exposure during pregnancy alters fetomaternal trafficking, leading to retention of fetal cells in the maternal lung and a concomitant decrease in the maternal liver. It can be assumed that this model of a chronic, very common lung injury is comparable to other chronic mild injuries in pregnancy, like inflammation due to chorioamnionitis.
